# Excellent Reproducibility of Synthetic Extracellular Volume Without Blood Extraction Across Different Cardiomyopathies Using Published Regression Models

**DOI:** 10.3390/jcdd13010034

**Published:** 2026-01-07

**Authors:** Jeong W. Choi, Sylvia Biso, Jonathan Weber, Karli Pipitone, Shibu Philip, Omar K. Khalique

**Affiliations:** Division of Cardiovascular Imaging, St. Francis Hospital & Heart Center, Roslyn, NY 11576, USA

**Keywords:** extracellular volume, synthetic ECV, infiltrative cardiomyopathy, synthetic hematocrit

## Abstract

T1 mapping and extracellular volume (ECV) calculations in cardiac magnetic resonance (CMR) have the potential to identify early fibrosis that is not yet visible using late gadolinium enhancement; however, the need for same-day blood draws due to the temporal variations in hematocrit (Hct) limits the use of ECV. We aimed to determine the reproducibility of synthetic Hct and ECV using different published models among groups of subjects. Healthy subjects and those with diagnosed cardiac amyloidosis, sarcoidosis, and hypertrophic cardiomyopathy (HCM) scanned using a 1.5T scanner with native and post-contrast T1 maps and same-day Hct were included. Among 148 subjects, there was excellent reproducibility (all ICCs ~0.98) between synthetic and measured ECV across the six formulas, despite only modest reproducibility of synthetic/measured Hct (ICCs 0.52–0.66). The levels of accuracy predicting abnormal measured ECV were consistently excellent among the different synthetic ECV models. The difference in the CMR vendor used to generate models did not seem to affect the results of the comparisons. We conclude that synthetic ECV yielded excellent reproducibility compared with ECV calculated using measured hematocrit, possibly obviating the need for blood extraction in cardiac MRI settings without point-of-care Hct.

## 1. Introduction

Cardiac magnetic resonance imaging (CMR) has demonstrated a high ability to assess cardiac function and tissue structure. CMR parametric mapping allows for the visual assessment of changes in tissue parameters such as T1, T2, T2*, and extracellular volume (ECV) across a range of disease states and associated adverse remodeling [1]. ECV reflects extracellular changes, such as interstitial expansion from fibrosis, edema, or deposition of abnormal substances [1]; has been validated against histologic collagen volume fraction (CVF); and has correlated well with cardiovascular outcomes [2].

ECV, as a volume fraction, varies with vascular volume changes. To account for these changes, calculating ECV requires a serum hematocrit (Hct) assessment. The intra-individual and diurnal variations in one’s Hct require it to be drawn ideally on the same day of the scan [3]. This presents a logistical challenge for many and limits the widespread adoption of routine ECV assessment in the absence of on-site equipment. While reports suggest that Hct drawn beyond the same day may be acceptable among patients without severe anemia [4], a proposed alternative to blood-drawn conventional Hct is to calculate a synthetic Hct from T1 maps.

Iron in hemoglobin has paramagnetic effects, and Hct is found to have a linear relationship with 1/T1 values [5]. Using this relationship, there have been studies that used locally derived and validated regression analysis models to determine synthetic Hct in the calculation of a synthetic ECV [6]. Whereas synthetic Hct appears to have a moderate correlation to conventional Hct, synthetic ECV, on the other hand, has a strong correlation to conventional ECV [7,8,9]. Despite these findings, there have been concerns that the use of synthetic Hct and ECV may misclassify abnormal vs. normal ECV values in clinical settings [10,11].

A recent meta-analysis pooling results from 10 studies (4492 patients) examining various synthetic ECV formulas found excellent correlations with laboratory ECV but recommended that each institution develop site-specific formulas instead of selecting one that was previously published [12]. Given the various published approaches to synthetic ECV, there is a need to standardize the different predictive models and validate them across different clinical populations and risk profiles. This study aimed to compare the different synthetic models for 1.5T MRI scanners in terms of reproducibility between conventional and synthetic Hct and ECV in healthy patients, in those with amyloidosis, sarcoidosis, and hypertrophic cardiomyopathy (HCM). We also aimed to assess the impact of anemia and evaluate the potential for misclassifying patients as abnormal versus normal ECV using synthetic methods. We hypothesized that synthetic ECV will closely approximate conventionally measured ECV on average, with greater variability observed among anemic patients.

## 2. Materials and Methods

### 2.1. Patient Population

This study retrospectively enrolled patients who underwent CMR for a clinical indication at St. Francis Heart & Hospital Center (Roslyn, NY, USA) between 2013 and 2023. A waiver of informed consent was obtained by the St. Francis Institutional Review Board, and all the work described has been carried out in accordance with the Declaration of Helsinki. We included patients who underwent CMR with acquisitions of native and post-contrast T1 maps using the modified Look–Locker inversion recovery sequence (MOLLI) and had Hct obtained on the same day of the scan. Patients who had hemochromatosis, had any contraindication for cardiac MRI (contraindicated metallic device, allergy to gadolinium enhancement material, or claustrophobia), or incomplete image acquisition were excluded. Three groups of patients with clinically established cardiac amyloidosis, cardiac sarcoidosis, and hypertrophic cardiomyopathy from prior research studies were identified and included. A control group consisting of subjects who had normal cardiac MRI results and no relevant cardiovascular, hepatic, or diabetic medical history was also included. Cardiac amyloidosis patients were diagnosed by positive bone scintigraphy for ATTR amyloidosis and the appropriate hematologic work-up for AL amyloidosis. Cardiac sarcoidosis patients were diagnosed by cardiac positron emission tomography and established clinical criteria. Hypertrophic cardiomyopathy patients met the criteria for diagnosis as per the guidelines [13].

### 2.2. Hematocrit

Hct was collected following a standard procedure. After the patient was registered for the exam, they were brought into the MRI suite, asked to don a hospital gown, and subsequently seated in an upright position in the patient preparation area while a nurse or trained MRI technologist performed venipuncture to draw the blood specimen and place an intravenous line for contrast administration. The blood specimens were brought to the main hospital at noon or at the end of the day and processed using a Sysmex XE-5000 or XN-2000 (after August 2015) Automated Hematology Analyzer (Sysmex America, Inc., Lincolnshire, IL, USA).

### 2.3. Cardiac MR Image Acquisition and Analysis Protocol

Cardiac MRI was performed using either 1.5T Siemens Magnetom Avanto (2013–2019) or Sola (2019–2023) scanners (Siemens Healthineers, Malvern, PA, USA) using an 18-channel body coil and a 32-channel spine coil. Standard CMR protocols employed were in accordance with the Society for Cardiovascular Magnetic Resonance guidelines. These included localizers, scouts, and balanced steady-state free precession-based cine images. T1 maps were performed using bSSFP-based modified Look–Locker inversion recovery (MOLLI) sequence acquired at the basal, mid, and apical short axis of the left ventricle. T1 images were obtained in a (5s(3s)3s) scheme prior to contrast and a 4(1)3(1)2 scheme 10 min post contrast. A standard gadolinium-based contrast bolus (gadoterate meglumine, Dotarem, Guerbet LLC., Princeton, NJ, USA) was given at a dose of 0.15 mmol/kg. Standard imaging parameters were used: field of view—400 × 300 mm^2^; matrix—256 × 166; TR/TE—301.7/1.09 ms; acquisition window duration—302 ms (native), 380 ms (post-contrast); flip angle—35 degrees; and slice thickness—8 mm.

**Figure 1:**
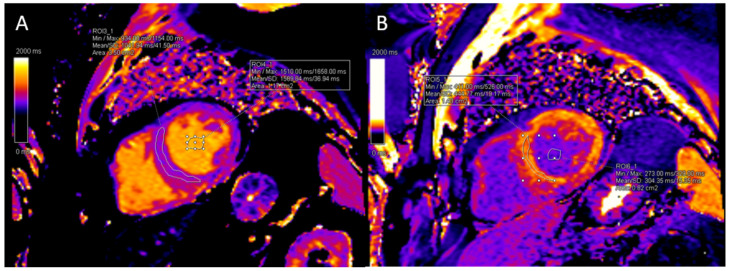
Example native (**A**) and post-contrast (**B**) T1-mapping images of a 76-year-old male subject without evidence of anemia.

The images were uploaded to a commercial software, Siemens syngo.via (Siemens Medical Solutions, Malvern, PA, USA), for offline analysis. Native and post-contrast myocardial T1 values were measured using freehand region-of-interest (ROI) on the mid-LV interventricular septum of a short-axis slice. Extra care was taken to make sure that the blood pool and LV fat were not included in the myocardial contours. Native and post-contrast blood-pool T1 values were obtained using a circular freehand ROI in the middle of the blood pool, avoiding papillary muscles. Figure 1 provides an example native and post-contrast T1-mapping acquisition and analysis.

### 2.4. Measured and Synthetic ECV

First, serum Hct was used to derive ECV percentage, calculated per society recommendations [2]:

$$ECV=\;\frac{\left(\frac{1}{{T1}_{{myo}_{post\;contrast}}}-\frac{1}{{T1}_{{myo}_{native}}}\right)}{\left(\frac{1}{{T1}_{{blood}_{post\;contrast}}}-\frac{1}{{T1}_{{blood}_{native}}}\right)}\times \left(100-hematocrit\right)$$

where myo = myocardium and blood = intracavitary blood pool. This was used as a measured standard to compare against synthetic ECV values calculated by replacing serum Hct with synthetic Hct in the ECV formula. Next, we selected six formulas from studies whose formulas were developed using MOLLI sequences on 1.5T MRI systems to estimate synthetic Hct and ECV, including those published by Treibel et al. [14], Fent et al. [6], Kammerlander et al. [15], Censi et al. [16], Opatril et al. [8], and Chen et al. [7] (Table 1).

### 2.5. Statistical Analyses

Continuous variables are reported as mean ± standard deviation and categorical variables as frequency (percent). Inter-class correlation coefficients were calculated according to standard guidelines [17] along with paired *t*-tests and Bland–Altman plots to compare the ability of synthetic Hct and ECV (based on the six synthetic Hct formulas) to replicate serum Hct and standard ECV. Replicability was assessed in the whole sample, within strata of CMR diagnosis groups, and between subjects whose measured Hct was considered anemic (Hct < 40 for males or <36 for females), regardless of diagnosis. We additionally examined the ability of each synthetic ECV to diagnose elevated (abnormal) ECV compared to ECV using the serum Hct. An ECV < 20% or >32% was considered abnormal based on current guidelines using a 1.5T Siemens MRI system using a MOLLI sequence [18]. All analyses were performed using SAS version 9.4 (SAS Institute, Inc., Cary, NC, USA), and plots were created using RStudio version 2025.09.2+418 (Posit Software, PBC, Boston, MA, USA).

## 3. Results

CMR studies were performed in 148 patients, including 36 (24%) normal control subjects, 36 (24%) patients with a diagnosis of cardiac amyloidosis, 20 (14%) with hypertrophic cardiomyopathy, and 56 (38%) with a diagnosis of cardiac sarcoidosis.

There were 31 (21%) non-control patients who were considered anemic (regardless of clinical diagnosis). Table 2 shows baseline clinical and CMR characteristics of the study sample. The mean age was 58 ± 14, and 39 (26%) were women. The mean native myocardial T1 time was 1022 ± 77 ms, and the mean native blood pool T1 time was 1571 ± 109 ms. The mean measured Hct was 41.5 ± 5%.

**Figure 2:**
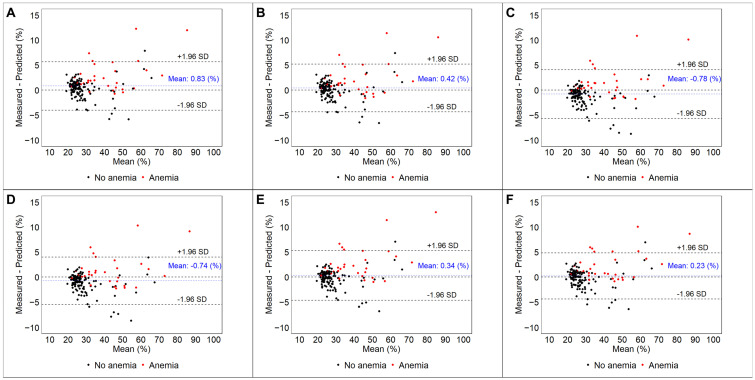
Bland–Altman plots among all subjects demonstrating reproducibility using six different synthetic ECV formulas, with stratification between anemic and non-anemic subjects: (**A**) Treibel et al. [14]; (**B**) Fent et al. [6]; (**C**) Kammerlander et al. [15]; (**D**) Censi et al. [16]; (**E**) Opatril et al. [8]; (**F**) Chen et al. [7].

Using previously published methods of deriving synthetic Hct and ECV, we applied the respective methods to our patient cohort (Table 3). All methods had low or modest ICCs with statistically significant differences between the measured and synthetic Hct in all but the methods by Opatril et al. and Chen et al. The Chen model had the highest ICC of 0.658. For synthetic ECV, all methods demonstrated excellent agreement with statistically significant (but smaller) differences between the measured and synthetic ECV in all but the Opatril and Chen methods. Synthetic ECV calculated by the Kammerlander and Censi methods slightly overestimated measured ECV, while all other methods slightly underestimated measured ECV. Bland–Altman plots demonstrating the reproducibility of synthetic vs. measured ECV among all methods, and also stratified by the presence of anemia, are displayed in Figure 2.

Among anemic patients (N = 31), overall agreement between synthetic Hct and the reference standard was poor but similar across synthetic methods, with ICCs ranging from 0.24 to 0.38 (Table 4A,B). Synthetic methods systematically overestimated Hct (falsely appearing more normal), with mean differences ranging from 1.9 ± 4.2% (using Censi’s model) to 4.4 ± 4.3 (using Treibel’s model). This was not the case for subjects without anemia (N = 117) who demonstrated moderate reproducibility of synthetic Hct (ICC range 0.36–0.58). The impact of anemia was less apparent for synthetic ECV with excellent and similar reproducibility (ICCs range 0.96–0.98 in both strata). However, anemic subjects’ synthetic ECV remained underestimated (falsely appearing more normal) by a range of −1.2 ± 3% (Censi) to −2.9 ± 3.2% (Treibel) compared to reference ECV.

Disease group-specific reproducibility comparisons were examined between each synthetic Hct and ECV method (Table 5). Reproducibility of Hct among disease groups was poor to moderate overall, with hypertrophic cardiomyopathy patients consistently demonstrating the worst reproducibility across all synthetic methods. The methods created by Kammerlander et al. and Chen et al. consistently produced the lowest and highest ICCs among the disease groups, respectively. The range of ICCs comparing the reproducibility of synthetic ECV methods was much narrower; there was not one method that consistently performed better or worse than other methods among the different disease groups.

We examined the reproducibility and accuracy of diagnosing abnormal ECV using the synthetic ECV methods (Table 6). Reproducibility was high and very similar across methods, with Cohen’s Kappa statistic ranging from 0.81 to 0.83. Measures of diagnostic accuracy, including sensitivity (range 88–90%), specificity (range 92–95%), as well as positive (range 83–88) and negative (range 95–96) predictive values, were also very similar. There were between 10 and 12 cases misdiagnosed; the majority (5–8 cases) occurred as false-negative and 4–5 as false-positive cases.

## 4. Discussion

Our comparison of six different published methods of calculating synthetic ECV demonstrates similar overall reproducibility of measured ECV among all subjects and different disease groups, as well as diagnostic performance (92–93% accuracy), lending support to their implementation. Among anemic patients, systematic overestimation of Hct led to the underestimation of ECV by all synthetic models. Stratification by disease group did not highlight a best-performing model for synthetic ECV.

Parametric mapping (T1, T2, T2*) in CMR allows for the detection of subtle changes across the spectrum of health and disease and allows for the detection of both intracellular (T1, T2, T2*) and extracellular changes in the form of ECV. While the benefits have been affirmed by experts [2,19], guidelines [20], and consensus statements [21,22], the logistical inconvenience of a blood draw hematocrit remains a persistent barrier for many. To this end, various groups have leveraged the paramagnetic properties of hemoglobin (as demonstrated in R1) and have empirically derived a linear regression model to predict synthetic hematocrit to use in the calculation of synthetic ECV. We evaluated six published methods and found excellent reproducibility in the calculation of synthetic ECV for clinical applications. Our results also demonstrate excellent reproducible correlation between the measured and synthetic ECV across normal subjects, those with HCM, cardiac amyloidosis, and sarcoidosis (ICC 0.976–0.980), with minimal bias. Our work expands on the work performed by Chen et al., which demonstrated reliability in patients with heart failure with reduced ejection fraction (HFrEF) and amyloidosis by validating performance in HCM and cardiac sarcoidosis patients.

In our analysis, all synthetic ECV models demonstrated systematic underestimation in anemic patients, which is consistent with prior observations, reflecting a systematic overestimation of synthetic Hct [4,7]. This systematic overestimation of ECV appears to remain stable across the range of ECV values, suggesting a parallel relationship between normal/anemic subjects, which would be reflected in a change in y-intercept rather than slope of any synthetic model. Future work should attempt to examine changes in the y-intercept or slope in synthetic models developed separately using anemic versus non-anemic subjects.

It bears noting that, in contrast to the findings by Rauci et al. [10], we found that in our adult population, overall model performances were better (92–93% accuracy versus 63–77%), suggesting that synthetic ECV may be more robust in adults. There are a few considerations that may contribute to the observed difference. Heart rate variability can lead to significant inaccuracy in post-contrast T1 values [23], which may affect ECV calculation. Going further, pediatric studies may need to account for partial volume averaging, which may also affect T1 measurements and discrepant results from our findings. Finally, our study utilized a 32-channel radiofrequency coil, compared to an 8-channel one used by the Raucci group, which introduces not only a difference of 50% in signal-to-noise improvement, but also a shorter breath-hold.

Our work validates the recently published meta-analysis, which found excellent correlations between synthetic and laboratory ECV by pooling together results of 10 synthetic ECV studies, many of which are included in this report [12]. We surpassed the heterogeneous limitation of this meta-analysis by comparing six synthetic ECV methods among the same patients each time, external to the original publications, as well as among anemic and non-ischemic disease subgroups. We included all formulas in the meta-analysis that applied to our own laboratory setup up as well as an additional study (Fent et al. [6]) excluded from the meta-analysis.

The high correlation between measured ECV and synthetic ECV was maintained despite a systematic underestimation of Hct using a prediction model. As previously suggested, overall changes to ECV calculation are likely attenuated since four additional factors are included in the ECV formula, which remain unchanged when synthetic Hct is incorporated [12]. Additionally, prior work has suggested that since R1 is a direct measurement of the blood pool in the left ventricle, it may be more reliable than serum Hct measured from blood drawn from the peripheral vasculature [10,11,12,16,24].

There are a few limitations to note. This is a retrospective single-center observational study, suggesting that the results of this study should be considered hypothesis-generating. We were unable to validate the synthetic models with clinical outcomes due to the limited number of clinical outcomes that occurred in our sample. Our results did not include patients with iron overload states, such as hemochromatosis or thalassemia (in which the R1/Hct relationship is not as strong), which limits generalizability. Third, the relatively smaller anemic sample size provides less statistical power. It is unclear if the overall performance would be affected if there were a larger anemic sample size.

## 5. Conclusions

Taken as a whole, we found that the use of synthetic ECV is very reproducible and remains robust across various non-ischemic cardiomyopathies. Model performance is not as strong in patients with anemia; nevertheless, we believe that there is an established benefit of synthetic ECV for patients without anemia. Future research should work to standardize these published formulas to their appropriate populations of use and validate them with appropriate clinical outcomes.

## Figures and Tables

**Table 1:** Previously published synthetic Hct and ECV models.

Study	Sample Size	Scanner	Sequence	Notes on the Math Model Used	Formula
Treibel et al. [14]	All subjects (N = 427) Derivation (n = 214) Validation (n = 213)	Siemens	MOLLI and shMOLLI		$HCTsyn=\left(866.0\times \frac{1}{{T1}_{blood}}\right)-0.1232$
Fent et al. [6]	All subjects (N = 421) 1.5T (n = 203) 3T (n = 218)	Philips	MOLLI		$1.5T:HCTsyn=\left(922.6\times \frac{1}{{T1}_{blood}}\right)-0.1668$
Kammerlander et al. [15]	All subjects (N = 513) Derivation (n = 200) Validation (n = 313)	Siemens	MOLLI		HCTsyn=628.5×R1−0.002
Censi et al. [16]	All subjects (N = 165) Derivation (n = 83) Validation (n = 82)	Philips	MOLLI		$HCTsyn=\left(761.8\times \frac{1}{{T1}_{blood}}\right)-0.086$
Opatril et al. [8]	All subjects (N = 139)	Philips	MOLLI	Compared linear vs. reciprocal regression model (reciprocal model is better)	$HCTsyn=-0.047+\left(\frac{779}{{T1}_{blood}}\right)-\left(\frac{11.69}{{T1}_{blood\;(post)}}\right)$
Chen et al. [7]	All subjects (N = 1101) Derivation (n = 550) Validation (n = 551)	Philips (and 3T Philips)	MOLLI	Deming regression used over ordinary least squares regression (Ord reg underestimates the regression coefficient when the predictor variable is subject to measurement error)	Male LV: $HCTsyn=1319\times \left(\frac{1}{{T1}_{blood}}\right)-0.4318$ Male RV: $HCTsyn=1137\times \left(\frac{1}{{T1}_{blood}}\right)-0.3212$ Female LV: $HCTsyn=1048\times \left(\frac{1}{{T1}_{blood}}\right)-0.2630$ Female RV: $HCTsyn=897.9\times \left(\frac{1}{{T1}_{blood}}\right)-0.1718$ LV multiple regression equation: * $HCTsyn=816.325\times \left(\frac{1}{{T1}_{blood}}\right)+0.024\times Sex-0.094\times Scanner-0.027$

Abbreviations: Hct—hematocrit; HCTsyn—synthetic hematocrit; LV—left ventricle; MOLLI—modified Look–Locker inversion recovery; RV—right ventricle; R1—blood relaxivity; shMOLLI, shortened modified Look–Locker inversion recovery; T1_blood_—native blood pool T1; T1_blood (post)_—post-contrast blood pool T1; * Substitute “1” if the “Sex” was male and “0” for female, substitute “1” if Scanner field strength was 1.5T and “0” for 3T.

**Table 2:** Baseline characteristics (N = 148).

Demographics	Mean ± SD, N (%)
Female	39 (26)
Age (years)	58 ± 14
BSA (m^2^)	
Cardiac amyloidosis	36 (24)
Hypertrophic cardiomyopathy	20 (14)
Cardiac sarcoidosis	56 (38)
Clinical	
LV EDV (mL/m^2^)	85 ± 23
LV ESV (mL/m^2^)	41 ± 22
LV mass index (g/m^2^)	67 ± 24
Stroke volume (mL)	89 ± 26
LVEF (%)	53 ± 11
Native myocardial T1 time (ms)	1022 ± 77
Native LV blood T1 time (ms)	1571 ± 109
Measured Hct (%)	41.5 ± 5

**Table 3:** Reproducibility of synthetic hematocrit and extracellular volume among all subjects (N = 148).

Synthetic Method	Measured Mean ± SD	Synthetic Mean ± SD	Mean Difference	*p*-Value	ICC (95% LL, UL)
Hematocrit					
Treibel et al. [14]	41.5 ± 5.0	43.1 ± 3.8	1.5 ± 3.8	<0.001	0.600 (0.499, 0.685)
Fent et al. [6]	41.5 ± 5.0	42.3 ± 4.0	0.8 ± 3.8	0.0165	0.636 (0.534, 0.719)
Kammerlander et al. [15]	41.5 ± 5.0	40 ± 2.7	−1.6 ± 3.8	<0.001	0.516 (0.424, 0.598)
Censi et al. [16]	41.5 ± 5.0	40.1 ± 3.3	−1.4 ± 3.8	<0.001	0.575 (0.476, 0.659)
Opatril et al. [8]	41.5 ± 5.0	42 ± 3.4	0.5 ± 3.8	0.1249	0.600 (0.500, 0.683)
Chen et al. [7]	41.5 ± 5.0	41.9 ± 4.1	0.3 ± 3.8	0.274	0.658 (0.560, 0.738)
Extracellular volume					
Treibel et al. [14]	31.5 ± 12.3	30.7 ± 11.5	−0.8 ± 2.5	<0.001	0.976 (0.968, 0.982)
Fent et al. [6]	31.5 ± 12.3	31.1 ± 11.7	−0.4 ± 2.4	0.0375	0.979 (0.971, 0.984)
Kammerlander et al. [15]	31.5 ± 12.3	32.3 ± 11.9	0.8 ± 2.5	<0.001	0.977 (0.968, 0.983)
Censi et al. [16]	31.5 ± 12.3	32.2 ± 11.9	0.7 ± 2.4	<0.001	0.978 (0.97, 0.984)
Opatril et al. [8]	31.5 ± 12.3	31.2 ± 11.4	−0.3 ± 2.5	0.108	0.977 (0.969, 0.983)
Chen et al. [7]	31.5 ± 12.3	31.3 ± 11.6	−0.2 ± 2.4	0.2436	0.980 (0.973, 0.986)

Abbreviations: SD—standard deviation; ICC—inter-class correlation coefficient; LL—lower limit; UL—upper limit.

**Table 4:** (**A**) Reproducibility of synthetic hematocrit and extracellular volume among anemic subjects (N = 31). (**B**) Reproducibility of synthetic hematocrit and extracellular volume among non-anemic subjects (N = 117).

(**A**)					
**Synthetic Method**	**Measured** **Mean ± SD**	**Synthetic** **Mean ± SD**	**Mean Difference**	***p*** **-Value**	**ICC (95% LL, UL)**
Hematocrit					
Treibel et al. [14]	34.9 ± 4.5	39.3 ± 3.0	4.4 ± 4.3	<0.001	0.237 (0.023, 0.430)
Fent et al. [6]	34.9 ± 4.5	38.3 ± 3.2	3.4 ± 4.3	<0.001	0.291 (0.034, 0.511)
Kammerlander et al. [15]	34.9 ± 4.5	37.3 ± 2.2	2.4 ± 4.1	0.0033	0.273 (0.037, 0.480)
Censi et al. [16]	34.9 ± 4.5	36.8 ± 2.7	1.9 ± 4.2	0.0163	0.327 (0.048, 0.559)
Opatril et al. [8]	34.9 ± 4.5	38.9 ± 2.6	4 ± 4.2	<0.001	0.226 (0.019, 0.415)
Chen et al. [7]	34.9 ± 4.5	38.3 ± 3.4	3.4 ± 3.9	<0.001	0.381 (0.129, 0.587)
Extracellular volume					
Treibel et al. [14]	43.2 ± 15.6	40.4 ± 14.2	−2.9 ± 3.2	<0.001	0.959 (0.921, 0.979)
Fent et al. [6]	43.2 ± 15.6	41.0 ± 14.5	−2.2 ± 3.2	<0.001	0.967 (0.935, 0.984)
Kammerlander et al. [15]	43.2 ± 15.6	41.7 ± 14.6	−1.6 ± 3.0	0.0078	0.975 (0.949, 0.987)
Censi et al. [16]	43.2 ± 15.6	42.0 ± 14.7	−1.2 ± 3.0	0.0295	0.977 (0.952, 0.989)
Opatril et al. [8]	43.2 ± 15.6	40.6 ± 14.1	−2.7 ± 3.3	<0.001	0.960 (0.923, 0.979)
Chen et al. [7]	43.2 ± 15.6	41.0 ± 14.5	−2.2 ± 2.8	<0.001	0.972 (0.945, 0.986)
(**B**)					
**Synthetic Method**	**Measured Mean ± SD**	**Synthetic Mean ± SD**	**Mean Difference**	***p*** **-Value**	**ICC (95% LL, UL)**
Hematocrit					
Treibel et al. [14]	43.3 ± 3.5	44.0 ± 3.3	0.7 ± 3.3	0.0167	0.514 (0.369, 0.634)
Fent et al. [6]	43.3 ± 3.5	43.4 ± 3.5	0.1 ± 3.4	0.8385	0.527 (0.381, 0.647)
Kammerlander et al. [15]	43.3 ± 3.5	40.7 ± 2.4	−2.6 ± 3.0	<0.001	0.356 (0.241, 0.462)
Censi et al. [16]	43.3 ± 3.5	41.0 ± 2.9	−2.3 ± 3.1	<0.001	0.410 (0.282, 0.524)
Opatril et al. [8]	43.3 ± 3.5	42.9 ± 3.1	−0.5 ± 3.1	0.1171	0.541 (0.401, 0.657)
Chen et al. [7]	43.3 ± 3.5	42.8 ± 3.8	−0.5 ± 3.3	0.1218	0.575 (0.440, 0.684)
Extracellular volume					
Treibel et al. [14]	28.4 ± 9.1	28.1 ± 9.1	−0.3 ± 1.9	0.1127	0.978 (0.968, 0.985)
Fent et al. [6]	28.4 ± 9.1	28.4 ± 9.3	0.1 ± 2.0	0.7525	0.977 (0.967, 0.984)
Kammerlander et al. [15]	28.4 ± 9.1	29.8 ± 9.7	1.4 ± 1.9	<0.001	0.969 (0.956, 0.978)
Censi et al. [16]	28.4 ± 9.1	29.6 ± 9.6	1.3 ± 1.9	<0.001	0.970 (0.958, 0.979)
Opatril et al. [8]	28.4 ± 9.1	28.7 ± 9.2	0.3 ± 1.9	0.0961	0.979 (0.969, 0.985)
Chen et al. [7]	28.4 ± 9.1	28.7 ± 9.2	0.3 ± 1.9	0.0855	0.977 (0.968, 0.984)

Abbreviations: SD—standard deviation; ICC—inter-class correlation coefficient; LL—lower limit; UL—upper limit.

**Table 5:** Reproducibility * of synthetic hematocrit and extracellular volume among disease groups.

	Normal Subjects (N = 36)		Hypertrophic Cardiomyopathy (N = 20)		Amyloidosis (N = 36)		Sarcoidosis (N = 56)	
	Mean Difference	ICC (95% LL, UL)	Mean Difference	ICC (95% LL, UL)	Mean Difference	ICC (95% LL, UL)	Mean Difference	ICC (95% LL, UL)
Hematocrit								
Treibel et al. [14]	−0.2 ± 3.2	0.605 (0.384, 0.760)	−1.3 ± 4.5	0.348 (−0.023, 0.634)	−1.8 ± 4.6	0.607 (0.400, 0.755)	−2.2 ± 3.2	0.547 (0.362, 0.690)
Fent et al. [6]	0.5 ± 3.2	0.615 (0.389, 0.772)	−0.5 ± 4.5	0.375 (−0.021, 0.669)	−0.9 ± 4.6	0.648 (0.440, 0.790)	−1.6 ± 3.2	0.593 (0.405, 0.734)
Kammerlander et al. [15]	2.8 ± 3.3	0.382 (0.205, 0.536)	1.7 ± 4.5	0.27 (−0.019, 0.518)	0.6 ± 4.9	0.534 (0.358, 0.673)	1.3 ± 2.9	0.556 (0.377, 0.694)
Censi et al. [16]	2.7 ± 3.3	0.443 (0.243, 0.606)	1.6 ± 4.5	0.312 (−0.022, 0.583)	0.9 ± 4.7	0.595 (0.400, 0.738)	0.9 ± 3.0	0.612 (0.425, 0.748)
Opatril et al. [8]	1.0 ± 3.1	0.596 (0.386, 0.748)	−0.2 ± 4.4	0.359 (0.003, 0.634)	−1.4 ± 4.8	0.579 (0.376, 0.730)	−0.9 ± 3.1	0.607 (0.416, 0.746)
Chen et al. [7]	1.6 ± 3.1	0.636 (0.413, 0.787)	0.1 ± 4.3	0.449 (0.053, 0.723)	−0.9 ± 4.4	0.687 (0.496, 0.815)	−1.4 ± 3.2	0.608 (0.420, 0.746)
Extracellular volume								
Treibel et al. [14]	0.1 ± 1.5	0.854 (0.728, 0.925)	0.6 ± 2.3	0.816 (0.574, 0.927)	1.6 ± 4.0	0.936 (0.884, 0.965)	1.0 ± 1.4	0.799 (0.689, 0.873)
Fent et al. [6]	−0.3 ± 1.5	0.851 (0.724, 0.922)	0.2 ± 2.3	0.823 (0.586, 0.930)	0.9 ± 4.0	0.943 (0.896, 0.969)	0.7 ± 1.4	0.825 (0.721, 0.892)
Kammerlander et al. [15]	−1.3 ± 1.6	0.752 (0.582, 0.859)	−0.9 ± 2.3	0.795 (0.539, 0.917)	−0.5 ± 4.1	0.942 (0.891, 0.970)	−0.6 ± 1.3	0.839 (0.748, 0.899)
Censi et al. [16]	−1.3 ± 1.6	0.768 (0.606, 0.869)	−0.9 ± 2.3	0.800 (0.549, 0.919)	−0.7 ± 4.0	0.946 (0.898, 0.972)	−0.4 ± 1.3	0.854 (0.765, 0.911)
Opatril et al. [8]	−0.5 ± 1.5	0.841 (0.708, 0.917)	0.0 ± 2.3	0.822 (0.585, 0.930)	1.2 ± 4.2	0.935 (0.882, 0.965)	0.4 ± 1.3	0.842 (0.749, 0.903)
Chen et al. [7]	−0.8 ± 1.5	0.832 (0.697, 0.910)	−0.1 ± 2.2	0.837 (0.616, 0.936)	0.9 ± 3.7	0.951 (0.910, 0.973)	0.6 ± 1.4	0.836 (0.737, 0.900)

* displayed as mean difference between serum and synthetic hematocrit and inter-class correlation coefficients. Abbreviations: ICC—inter-class correlation coefficient; LL—lower limit; UL—upper limit.

**Table 6:** Accuracy in predicting abnormal extracellular volume from synthetic models.

Synthetic Method	Accuracy	Sens	Spec	PPV	NPV	Cohen’s Kappa
Treibel et al. [14]	93	88	95	88	95	0.83 (0.73, 0.93)
Fent et al. [6]	93	90	93	84	96	0.82 (0.72, 0.92)
Kammerlander et al. [15]	92	90	92	83	96	0.81 (0.70, 0.91)
Censi et al. [16]	92	90	92	83	96	0.81 (0.70, 0.91)
Opatril et al. [8]	93	88	95	88	95	0.83 (0.73, 0.93)
Chen et al. [7]	92	90	92	83	96	0.81 (0.70, 0.91)

Abbreviations: Sens—sensitivity; Spec—specificity; PPV—positive predictive value; NPV—negative predictive value.

## Data Availability

The data is available by reasonable request made to the corresponding author.

## Abbreviations

The following abbreviations are used in this manuscript:

CMR	Cardiovascular magnetic resonance
ECV	Extracellular volume fraction
HCM	Hypertrophic cardiomyopathy
ICC	Intraclass correlation coefficient
Hct	Hematocrit
MOLLI	Modified Look–Locker inversion recovery
bSSFP	Balanced steady state free precession
HCTsyn	Synthetic hematocrit
HFrEF	Heart failure with reduced ejection fraction
NPV	Negative predictive value
PPV	Positive predictive value
LV	Left ventricle
RV	Right ventricle
EDV	End-diastolic volume
ESV	End-systolic volume
ROI	Region of interest
Myo	Myocardium
